# Case report: A novel mutation of the CAPN3 gene in a Chinese family with limb-girdle muscular dystrophy type 2A

**DOI:** 10.3389/fgene.2024.1410727

**Published:** 2024-08-12

**Authors:** Wanjun Feng, Yanyan Cao, Ruolin Ren, Xiaohui Yang, Chunyan Cao, Hongwei Jiang, Ganqin Du

**Affiliations:** The First Affiliated Hospital, College of Clinical Medicine of Henan University of Science and Technology, Luoyang, China

**Keywords:** limb-girdle muscular dystrophy type 2A, CAPN3, progressive muscle weakness and wasting, gene splicing, autosomal recessive disorder

## Abstract

Limb-girdle muscular dystrophy type 2A (LGMD R1 Calpain 3-Related, LGMD2A/R1), an autosomal recessive disorder, is characterized by progressive muscle weakness with a prominent presentation in the proximal limb girdle muscles. LGMD2A/R1, which is caused by variants in calcium-activated neutral proteinase 3 (*CAPN3*), is the most common. The present study aimed at identifying the clinically significant variants in a Chinese family with LGMD2A/R1 and exploring the genotype–phenotype correlations. Clinical symptoms, laboratory findings, and physical examinations were obtained. Genomic DNA was extracted from the peripheral blood samples of this family. Whole-exome sequencing (WES) and Sanger sequencing were used to explore and validate the pathogenic genes. In this study, the proband and his sister, who had two identical mutations in the *CAPN3* gene sequence, exhibited diverse clinical features, including disease onset and progression. The mutation c.2120 A>G (p. D707G) is pathogenic and has been reported in the Human Gene Mutation Database (HGMD) and the ClinVar database. c.1783-72 C>G may be a novel pathogenic mutation of LGMD2A/R1 based on the American College of Medical Genetics (ACMG) guidelines, which widens the gene variant pool in *CAPN3* and improves diagnosis and genetic counseling.

## 1 Introduction

Limb-girdle muscular dystrophy (LGMD) is a genetically inherited disease, classified into autosomal dominant (AD-LGMD) and autosomal recessive (AR-LGMD) (Bushby, 1995). LGMD mainly affects the skeletal muscle, which progressively leads to the weakness of proximal muscle by loss of muscle fibers (Fanin and Angelini, 2015). Limb-girdle muscular dystrophy type 2A (LGMD R1 Calpain 3-Related, LGMD2A/R1), a prevalent form in many countries, is mainly caused by the calcium-activated neutral proteinase 3 (*CAPN3*) gene mutations (Fanin et al., 2009). *CAPN3* is a calcium-dependent cysteine protease responsible for assembling and remodeling the sarcomere, regulating calcium outflow from the sarcoplasmic reticulum, and repairing sarcolemma (Straub et al., 2018).

Diagnosing LGMD2A/R1 is difficult due to laboratory examination results and clinical manifestations that overlap other LGMDs (Fanin et al., 2009). Furthermore, the clinical manifestations of LGMD2A/R1 are highly variable in onset of age, degree of muscular weakness, and disease progression. Genotype–phenotype correlation is quite challenging due to the genetic heterogeneity of *CAPN3* and variations in clinical manifestations. Thus, the LGMD2A/R1 remains to be explored.

In the present study, we reported two siblings diagnosed as LGMD2A/R1. The siblings exhibited diverse clinical features with two identical mutations in the *CAPN3* gene sequence, one of which has been reported. The novel mutation c.1783-72 C>G may be pathogenic.

## 2 Case report and methods

### 2.1 Subjects

In this study, the clinical and genetic data of a Chinese family were collected. The case study was approved by the Ethics Committee of the First Affiliated Hospital and the College of Clinical Medicine of Henan University of Science and Technology, Luoyang, China (ethical approval number: 2022-03-B127). Informed consent was acquired from the patient (or relative/guardian) AND that patient (or relative/guardian) consented to the publishing of all images, clinical data, and other data included in the manuscript.

The proband (a 33-year-old male) was born to non-consanguineous Chinese parents. He presented with progressive weakness, decreased stride frequencies, increased unsteadiness in both lower limbs, and, at the age of 15, needed knee support while standing. Over time, he began to fall to the ground frequently and required help to rise. Three years ago, he could not walk, comb his hair, or get dressed unaided. The weakness and atrophy of the proximal limbs, a wide-based gait, hyperlordosis, scapular winging, and anterior pelvic tilt were found through physical examination. The strength was measured with the Medical Research Council muscle testing/MRC scale (MacAvoy and Green, 2007): deltoid 2/5, biceps/triceps 3/5, quadriceps and biceps femoris 3/5, and gluteus maximus 2/5. The facial and bulbar muscles were not involved. The cardiovascular and respiratory systems were normal. At 16 years of age, his blood analysis results showed elevated creatine kinase levels of 3,000 U/L (normal value < 150 U/L), which decreased to 1,700 U/L at 21. Electromyography (EMG) showed severe generalized myopathy with low-amplitude short-duration motor units. Nerve conduction was spared. The electrocardiogram (ECG) and ultrasound cardiogram (UCG) results were normal. He is now wheelchair-dependent and unable to take care of himself. Due to regular follow-up, the proband has a stable mood and sleeps well. The patient was treated with oral inosine and functional exercise. He consequently developed concomitant hyperthyroidism, and he was put on oral propylthiouracil tablets.

**FIGURE 1:**
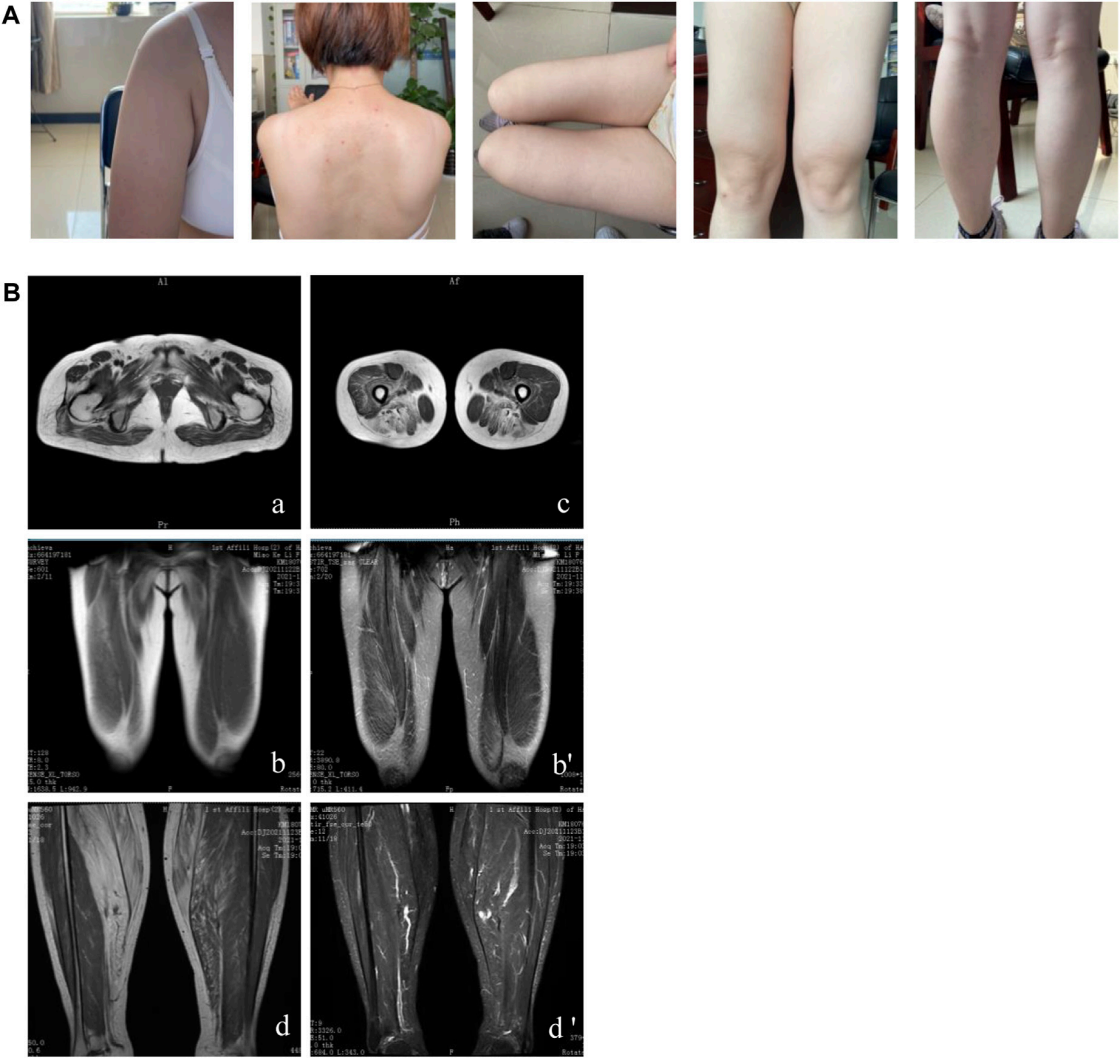
Clinical features and muscular MRI of patient II-3. **(A)** Winged scapulae, atrophy of the deltoid, inner muscles of the thigh and right calf, and left calf pseudo-hypertrophy are shown. **(B)** The affected glutei/iliopsoas muscles **(A)**, vastus lateralis/gracilis **(B)**, adductors/biceps femoris/semitendinosus and semimembranosus muscles **(C)**, and gastrocnemius/soleus **(D)** were presented through muscular MRI.

The sister of the proband was a 36-year-old woman. She complained of paroxysmal myalgia after exercise and hyperCKemia for 7 years. Progressively, she presented with proximal lower limb weakness. Her physical examination showed bilateral atrophy of the proximal lower limbs, scapular winging, bilateral calf hypertrophy, and slight weakness (the MRC scale: deltoid 4+/5, musculus biceps brachii 5−/5, forearm flexors 5/5, forearm extensor 4/5, iliopsoas and gluteus maximus 5−/5, pronator and supinator 5/5) and a positive Gower’s maneuver result. Her creatine kinase level was 1,800 U/L at the first evaluation and 3,000 U/L at the 1-month follow-up. EMG results showed features of myopathy. Within 3 years of follow-up, she had difficulty climbing the stairs and needed to hang on to the railing with both hands. On physical examination, the patient’s deltoid muscle, proximal thigh, and right calf were atrophic (Figure 1A). The proximal limb weakness progressively aggravated (the MRC scale: deltoid 4/5, musculus biceps brachii 4/5, forearm flexors 4/5, forearm extensor 3/5, iliopsoas and gluteus maximus 4-/5, pronator and supinator 5/5). Muscular MRI of the lower limb showed that the glutei/iliopsoas muscles, vastus lateralis/gracilis, adductors/biceps, femoris/semitendinosus and semimembranosus muscles, and gastrocnemius/soleus were affected (Figure 1B). Muscle fibers became thinner, accompanied by fat infiltration and edema. She had no sleeping or emotional disorder. She took vitamin E tablets and trimetazidine hydrochloride tablets orally. Meanwhile, she was prescribed functional training.

### 2.2 Muscle biopsies

Samples of the quadriceps femoris muscle of patient Ⅱ-3 were collected for muscle biopsies according to the conventional methods.

### 2.3 Whole-exome sequencing

Peripheral blood samples were collected from all the members of the family, and genomic DNA was extracted. Agilent SureSelect Human All Exon V6 reagent was used for capture and enrichment, and Illumina NovaSeq 6000 was applied for whole-exon gene sequencing in the proband. The results were aligned to the human reference genome hg19. Variations were annotated according to the American College of Medical Genetics and Genomics (ACMG) guidelines (Richards et al., 2015). Some databases, including the Human Gene Mutation Database (HGMD), ClinVar database, 1,000 Genomes, Exome Aggregation Consortium (ExAC), and genome Aggregation Database (gnomAD), were applied to determine the known or potential novel mutations. Predictions for the pathogenicity of mutations were performed with SIFT, PolyPhen-2, and MutationTaster.

### 2.4 Sanger sequencing

Sanger sequencing to predict and analyze the mutation splicing sites. The primers were designed to target *CAPN3* (Forward: 5′-CTC​CAA​GTG​CCT​TCT​GAA​TGA​CCA​CAG​GCG-3′; Reverse: 5′-CAT​CTC​GTA​GCT​GTT​GAT​GGT​GCC​GGA​CTG-3′). Genetic validation was performed at the Institute of Neurology, the First Affiliated Hospital of Fujian Medical University.

## 3 Results

### 3.1 Muscle biopsy histology results

**FIGURE 2:**
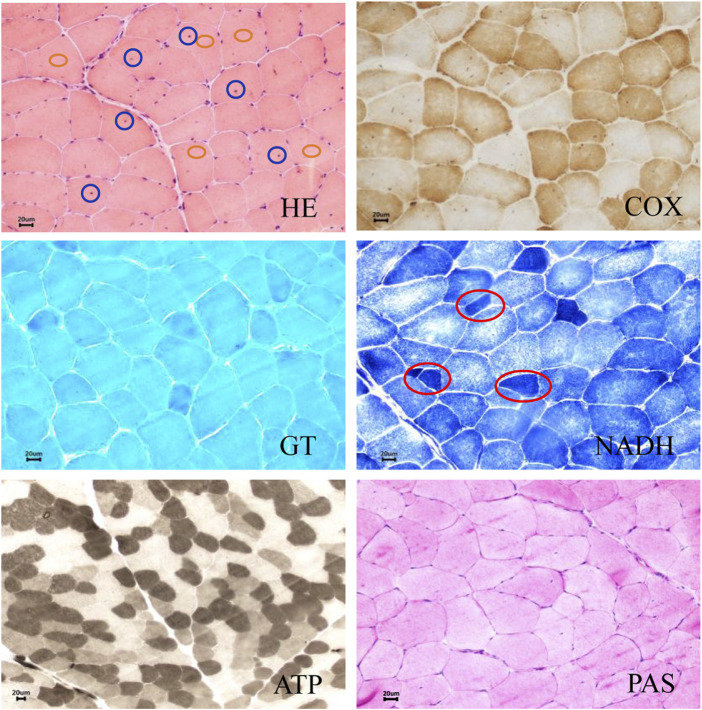
Muscle biopsy histology of patient II-3. Hematoxylin and eosin (H&E) show dystrophic features with fiber size variation, scattered small circular atrophic muscle fibers, and partial compensatory hypertrophic muscle fibers. Intranuclear migration was observed in some muscle fibers. The denatured necrotic muscle fibers were observed with a small amount of mononuclear phagocyte infiltration. Inflammatory cell infiltration was not observed in the connective tissue and adipose tissue. Adenosine triphosphatase (ATPase) staining showed that the fibers of type Ⅰ and type Ⅱ showed mosaic distribution, and no grouping phenomenon was observed. Gomori’s trichrome (GT) staining showed no typical or atypical ragged-red fibers (RRF) and rimmed vacuole (RV) in muscle fibers, and periodic acid-Schiff (PAS) staining showed no obvious glycogen increase in muscle fibers. No special changes were observed in succinic dehydrogenase (SDH) staining and cytochrome c oxidase (COX) staining. The blue circle represents intranuclear migration. The red circle represents muscle fiber atrophy. The orange circle represents muscle fiber hypertrophy. Scale bar: 20 µm.

Muscle biopsy histology was performed in patient Ⅱ-3 (Figure 2). Hematoxylin and eosin (H&E) show dystrophic features with fiber size variation, scattered small circular atrophic muscle fibers, and partial compensatory hypertrophic muscle fibers. Nuclear translocation was observed in some muscle fibers. The denatured necrotic muscle fibers were observed with a small amount of mononuclear phagocyte infiltration. The inflammatory cell infiltration was not observed in the connective tissue and adipose tissue. Adenosine triphosphatase (ATPase) staining indicated that the fibers of type Ⅰ and type Ⅱ showed mosaic distribution, and no grouping phenomenon was observed. Gomori’s trichrome (GT) staining showed no typical or atypical ragged-red fibers (RRF) and rimmed vacuole (RV) in muscle fibers, and periodic acid-Schiff (PAS) staining showed no obvious glycogen increase in muscle fibers. No special changes were observed in succinic dehydrogenase (SDH) staining and cytochrome c oxidase (COX) staining. We concluded that pathological changes were consistent with muscular dystrophy. Limb-girdle muscular dystrophy was suggested, and further gene sequencing was recommended.

### 3.2 Genetic screening results

**FIGURE 3:**
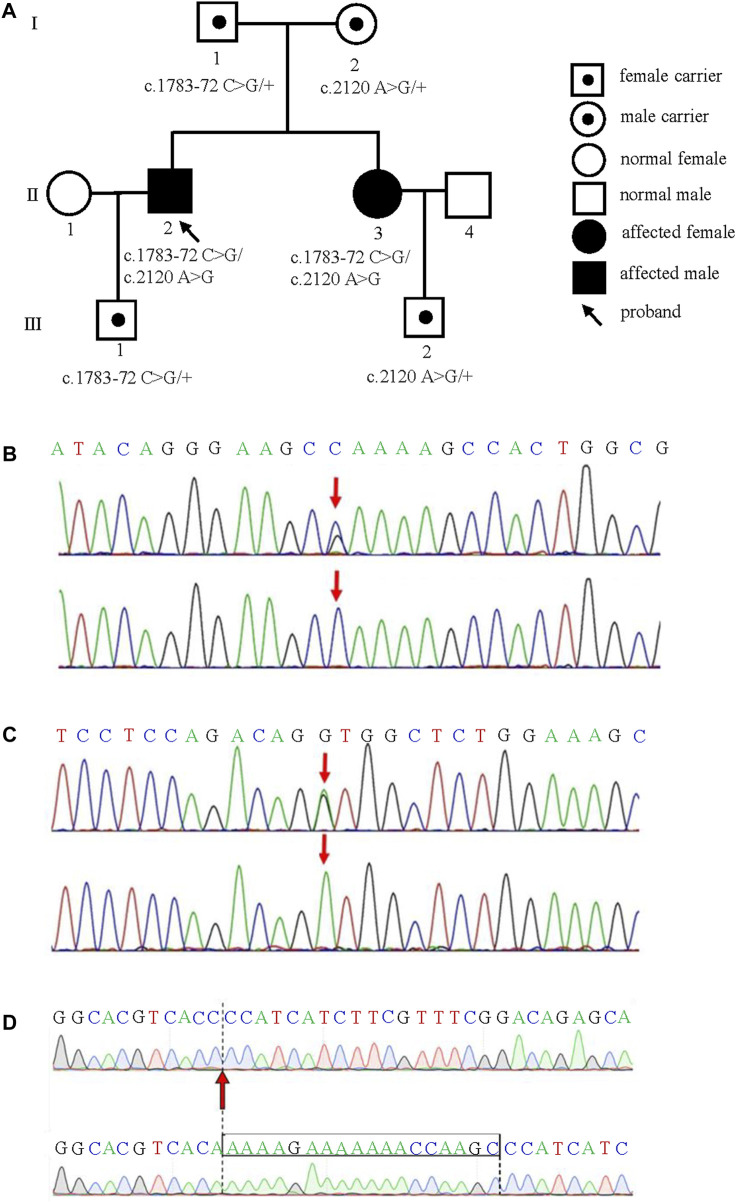
Compound heterozygous *CAPN3* variants causing limb-girdle muscular dystrophy type 2A in a Chinese family **(A)** The family pedigree of the limb-girdle muscular dystrophy type 2A. The proband and his sister carried the c.1783-72 C>G and c.2120 A>G (p. D707G) variants of CAPN3. The c.1783-72 C>G and c.2120 A>G (p. D707G) were inherited from the siblings’ father and mother, respectively. The mutation of c.1783-72 C>G **(B)** and c.2120 A>G (p. D707G) **(C)** in CAPN3 were obtained. The red arrow indicates the variant site. The normal sequence is shown below. **(D)** A skip of exon 2 near chr15-42698052-C-G (16 bp in total) in CAPN3 was detected by Sanger sequencing. The normal sequence is shown below.

The family pedigree was obtained (Figure 3A). The phenotypes of the proband and his sister were different (Table 1). However, the gene analysis results showed that two siblings possessed two identical mutations in the *CAPN3* gene that were derived from their parents (Table 2). The mutation c.1783-72 C>G from the father (Figure 3B) was not previously reported. According to the ACMG guidelines (Richards et al., 2015), its pathogenicity grade is PM3+PP1+PP3+PP4, which may be a “likely pathogenic” mutation. Another mutation, c.2120 A>G, from the mother, leading to amino acid changes (p.D707G) (Figure 3C), is the LGMD2A/R1 pathogenic mutation reported in the HGMD Professional and ClinVar databases.

**TABLE 1:** The phenotypes of the proband and his sister.

Phenotypes	Proband	Sister of proband
Onset age (years old)	15	29
Main sites of involvement	Girdle of the proximal limbs	Girdle of the proximal limbs
Time from disease onset to life dependence (years)	18	—
Wheelchair dependence	YES	NO
CK level	high	high
Comorbid diseases	Hyperthyreosis	—
Facial, cardiac, and respiratory involvement	—	—
Presence of scapular winging	YES	YES

**TABLE 2:** Candidate gene mutations of the proband.

Gene	Genetic	Nucleotide	Amino acid	1000 g	S|P|Mt|	Source of
	Mode	Change	Change	|ESP| ExAC*	Ms*	Variation
*CAPN3*	AR	c.A2120G	p.D707G	0.00|N/A|0.00	D|D|D|D	mother
*CAPN3*	AR	c.1783-72C>G	N/A	0.00|N/A|N/A	N/A	father
*NEFH*	AR,AD	c.C1036T	p.R346C	0.00|0.00|0.00	T|T|D|T	
*SYNE2*	AD	c.C5221T	p.H1741Y	N/A|N/A|0.00	T|T|N|T	

Abbreviations: AR, autosomal recessive; AD, autosomal dominant;

S|P|Mt|Ms: SIFT|POLYPHEN2|MUTATIONTASTER|METASVM.

SIFT: D, DELETERIOUS; T, TOLERATED;

POLYPHEN2: D, PROBABLY DAMAGING; P, POSSIBLY DAMAGING; B: BENIGN;

MUTATIONTASTER: A, DISEASE_CAUSING_AUTOMATIC; D, DISEASE_CAUSING; N;

METASVM: D, DELETERIOUS; T, TOLERATE.

### 3.3 Genetic validation results

The cDNA of the proband’s sister was amplified based on the result of the WES. Sanger sequencing showed that exon 2 had a skip (16 bp total) near the variant site chr15-42698052-C-G that validated the mutation c.1783-72 C >G detected by WES (Figure 3D).

## 4 Discussion

In the present study, the proband and his sister presented the typical clinical features of LGMDR1, including progressive proximal muscle weakness, calf hypertrophy, and winged scapula (Mahmood and Jiang, 1954). The sister of the proband may have benign clinical features, including paroxysmal myalgia after exercise and elevated serum creatine kinase level at onset. Although she developed muscular weakness, she was able to live independently. Meanwhile, the proband’s condition progressed rapidly, and he lived dependently. The age of onset, severity of the disease, and rate of progression significantly vary within this Chinese family, which suggests intra-familial phenotypic variability. This variability is consistent with reports of the same genetic variant in LGMDR1 that had a different clinical presentation (Schessl et al., 2008).

Great variation exists between the genotype and phenotype in LGMDR1. The significant differences in clinical manifestations among patients with limb-girdle muscular dystrophy with the same genetic mutations within one family can be attributed to several factors. First, genetic modifiers or variations in other genes may influence the expression and severity of the disease. These modifier genes can interact with the mutated gene, altering the disease phenotype. Second, environmental factors play a role. Different exposures to toxins, infections, diet, and physical activity levels can impact the progression and symptoms of the disorder. Epigenetic modifications, such as DNA methylation and histone acetylation, can also modify gene expression and contribute to the variability in clinical manifestations. Furthermore, individual variations in the immune system response and the body’s ability to cope with muscle damage and regeneration can lead to differences in the disease course and symptoms. In addition, stochastic events during development and aging processes may also contribute to the heterogeneity of the clinical picture among affected family members.

In our study, the mutation c. 2120 A>G is the LGMDR1 pathogenic mutation and has been reported in the HGMD Professional and ClinVar databases (Narihiro et al., 1999). The c.1783-72 C>G mutation has not been reported. According to ACMG (Richards et al., 2015), the pathogenicity grade of c.1783-72 C>G was PM3+PP1+PP3+PP4, suggesting that it may be a new pathogenic mutation of LGMDR1. The mutation c.1783-72 C>G could lead to exon 2 skipping identified by Sanger sequencing, which might affect the normal transcription of *CAPN3* in one chromosome. c.2120A > G (p. D707G) and c.1783-72 C> G performed a compound heterogenous mutation, which abolished the function of *CAPN3* to cause limb-girdle muscular dystrophy.

The underlying mechanisms of LGMDR1 have not been clear until now. In the skeletal muscle, the *CAPN3* encodes a Ca^2+^/Na^+^-dependent cysteine protease (calpain-3) that plays a key role in sarcomere formation, muscle remodeling, and the regulation of cytoskeleton (Tidball and Spencer, 2000; Yasuko et al., 2010). Except for its proteolytic activity, calpain-3 has a nonproteolytic structural function acting on Ca^2+^ handling in the skeletal muscle (Lasa-Elgarresta et al., 2019). A previous study reported that the knockout of *CAPN3* could damage the mitochondrial function in an animal model (Kramerova et al., 2009). Furthermore, mitochondrial deficiencies were found in LGMDR2A with calpain-3 mutations (El-Khoury et al., 2019). Thus, abnormality of the *CAPN3* gene may lead to defects of muscular fiber formation and weakness in LGMDR1 by affecting mitochondrial function and/or regulation of Ca^2+^ in muscle.

Some limitations exist. First, gene sequencing was not done on muscle tissue. Second, the proband could not participate in further imaging examination and muscle biopsy due to disability.

## 5 Conclusion

In conclusion, we reported two cases in which the same genotype of LGMDR1 had different phenotypes, including age of onset, disease severities, and rate of progression in a Chinese family. Two compound heterozygosity variants were found. c.1783-72 C > G mutation of *CANP3* may be a novel pathogenic mutation of LGMDR1. This broadens the understanding of LGMDR1.

## Data Availability

The datasets presented in this article are not readily available because of ethical and privacy restrictions. Requests to access the datasets should be directed to the corresponding author.
